# Immunopathogenic Mechanisms of Autoimmune Hepatitis: How Much Do We Know from Animal Models?

**DOI:** 10.3390/ijms17122007

**Published:** 2016-12-01

**Authors:** Urs Christen, Edith Hintermann

**Affiliations:** Pharmazentrum Frankfurt/ZAFES, Goethe University Hospital, Theodor-Stern Kai 7, 60590 Frankfurt am Main, Germany; hintermann@med.uni-frankfurt.de

**Keywords:** AIH immunopathogenesis, autoantibodies, T cells, cytokines, chemokines: CYP2D6 model

## Abstract

Autoimmune hepatitis (AIH) is characterized by a progressive destruction of the liver parenchyma and a chronic fibrosis. The current treatment of autoimmune hepatitis is still largely dependent on the administration of corticosteroids and cytostatic drugs. For a long time the development of novel therapeutic strategies has been hampered by a lack of understanding the basic immunopathogenic mechanisms of AIH and the absence of valid animal models. However, in the past decade, knowledge from clinical observations in AIH patients and the development of innovative animal models have led to a situation where critical factors driving the disease have been identified and alternative treatments are being evaluated. Here we will review the insight on the immunopathogenesis of AIH as gained from clinical observation and from animal models.

## 1. Clinical Features of Autoimmune Hepatitis

Autoimmune hepatitis (AIH) is a serious autoimmune liver disease that is characterized by a progressive destruction of the liver parenchyma and the development of chronic fibrosis [1,2,3,4,5]. An estimated 100,000 to 200,000 persons are currently affected by AIH in the USA [6,7] and, according to the World Health Organisation, AIH has an annual incidence of approximately 2 in 100,000 individuals and a prevalence 15 cases per 100,000 persons worldwide [8]. Thus, the prevalence and incidence of AIH is similar as for the two other major autoimmune liver diseases, primary biliary cholangitis (PBC) and primary sclerosing cholangitis (PSC). As in most autoimmune diseases, AIH has a female predominance (sex ratio, 3.6:1). It occurs in children and adults of all ages and affects several ethnic groups [9]. Two variants of AIH have been postulated in the past. Both variants are associated with major histocompatibility complex (MHC) class I human leukocyte antigen (HLA)-B8 and with MHC class II HLA-DR3 (DRB1*03:01). In addition, AIH type 1 is also associated with *HLA-DR4* (DRB1*04:01), whereas AIH type 2 is associated with *HLA-DR7* (DRB1*07:01) and *HLA-DQ2* (DQB1*02:01) [10,11,12,13,14,15,16]. A recent genome-wide association study (GWAS) on Dutch and German patients confirmed DRB1*03:01 and DRB1*04:01 as the primary and secondary susceptibility loci for AIH type 1 [17]. However, distinctive susceptibility variants have been reported for different ethnic groups (see [16] for a more detailed listing of HLA associations). Interestingly, the HLA haplotype seems to also influence the course of the disease: patients carrying the *HLA-B8* allele develop a more severe inflammation and are more likely to have a relapse after treatment. The presence of *HLA-DR3* is associated with a lower probability for remission and a higher relapse frequency as well as a frequent requirement for liver transplantation [18]. In addition, patients carrying DRB1*03:01 generate higher immunoglobulin G levels [19]. In contrast, individuals with *HLA-DR4* display a higher rate of complete remissions alongside a lower frequency of cirrhosis and are thus associated with a more favorable clinical outcome [20].

In general, the clinical spectrum of AIH ranges from asymptomatic to severe, with symptoms that are similar to those found in acute viral hepatitis or fulminant hepatic failure [3,21,22]. Thus, the diagnosis of AIH has been and still is challenging and depends on several factors including histological features as well as serum biomarkers, such as specific autoantibodies. The key histological features of AIH is the presence of an interface hepatitis/piecemeal necrosis affecting patches of hepatocytes characterized by plasmacytosis (infiltrating plasma cells), hepatocyte rosetting and emperipolesis [3,5,21]. According to the revised and simplified scoring system of the International AIH Group (IAIHG) [23], one of the core diagnostic criteria of AIH and its subtypes is the presence of specific antibodies to particular liver autoantigens [24,25]. Historically, AIH type 1 has been characterized by the presence of anti-nuclear (ANA) and/or anti-smooth muscle (SMA) autoantibodies, whereas type 1 liver/kidney microsomal autoantibodies (LKM-1) have been considered as the hallmark of AIH type 2 [3,9,21,26]. However, recently, such a classification has been questioned since patients with type 1 and type 2 AIH share the same clinical phenotype [27]. In addition, in some patients, the autoantibody profile changed from one subtype to another over time. AIH type 2 might as well constitute an early form of AIH appearing in younger patients who later during disease convert to an AIH type 1 phenotype. One of the most thoroughly characterized autoantigens is the 2D6 isoform of the large cytochrome P450 enzyme family (CYP2D6) that is recognized by LKM-1 antibodies and was identified in the late 1980s [28,29]. The majority of patients carry LKM-1 antibodies that recognize an immunodominant region spanning aa 256–269 [30,31]. However, reactivity of LKM-1 antibodies to several other CYP2D6 epitopes has been detected in various proportions of patients’ sera (reviewed in [25]). Importantly, CYP2D6-specific cluster of differentiation (CD) 4 and CD8 T cells were found in the blood and the liver of AIH patients [32,33].

## 2. Current Treatment

Due to the autoimmune nature of the disease, the traditional standard therapy of AIH is a glucocorticoid treatment with prednisone/prednisolone alone or in combination with azathioprine [5,22,34]. The goal of the therapy is to induce AIH remission indicated by a normalization of the serum aminotransferase levels and a reduction of the hypergammaglobulinemia (see a more detailed review on the clinical treatment of AIH by Mann et al. [5]). Alternative treatments have been introduced, in particular for treatment of AIH relapses after corticosteroid withdrawal. It has been demonstrated that the next-generation glucocorticoid budesonide and the calcineurin inhibitors cyclosporine A and tacrolimus improve the outcome of AIH [5,22,34]. Another promising drug is the immunosuppressant cytostatic drug mycophenolate mofetil that has been shown to be safe and effective as first-line or rescue therapy in inducing and maintaining remission [35]. Although the majority of individuals indeed achieve a remission during standard therapy, adults rarely achieve resolution of their laboratory and liver tissue abnormalities in less than 12 months and withdrawal of therapy after two years leads to relapse in 85% of cases [9]. In addition, long-term therapies of AIH carry the risk of significant steroid-specific and azathioprine-related side effects. Recently, small preliminary studies have been performed, evaluating more specific treatments, including the administration of anti-CD20 antibodies (i.e., rituximab) and tumor necrosis factor α (TNFα)-neutralizing biologics such as infliximab [36,37]. The outcome of these studies, namely a reduction of inflammation, is promising. However, due to the low number of treated patients (<10) these data should be considered with caution. In addition, patients treated with infliximab also experienced an increased risk of opportunistic infections [36].

Overall, it is somewhat surprising that the therapy of AIH is still largely restricted to a traditional immunosuppressive regimen including glucocorticoids and cytostatic drugs, although some of the major target antigens have been characterized even on the level of specific epitopes. The problem is that the etiology and the detailed immunopathogenic mechanisms of AIH are still only poorly understood. One major reason for this lack of comprehension is the fact that, in the past, there were not many reliable animal models available that could have answered the central questions of how tolerance to self-components, such as CYP2D6 and other molecules, is broken and what mechanisms subsequently promote and maintain the chronic inflammation and liver fibrosis in AIH.

## 3. Mechanistic Insight from Clinical Observations

The presence of liver autoantigen-specific antibodies and T cells led to the perception of AIH as an autoimmune disease affecting the liver in an organ-specific fashion. A variety of autoantibodies have been found in the past and their reactivity to liver autoantigens has been characterized to the level of individual immunodominant epitopes that are recognized by the majority of AIH patients [24,25]. Although there is a considerable overlap in the specificity of autoantibodies found in patients diagnosed with AIH, PBC and PSC [38], the presence of many autoantibodies are included in the overall diagnostics of AIH [23]. However, it is not clear if such autoantibodies also have a pathologic role. It has been found that the titers of autoantibodies correlate with other parameters that characterize disease severity, such as serum aminotransferases and histology [39], but this might also be an epiphenomenon of the exacerbated liver damage. Potentially, autoantibodies may induce cellular damage by different means, such as complement activation, fragment Fc receptor binding, and immune complex formation. Indeed, several observations suggest a certain pathogenic role for autoantibodies. First, isolated hepatocytes from AIH patients are often covered with antibodies [40]. Second, such hepatocytes decorated with autoantibodies are sensitive to cytotoxic killing by mononuclear cells [40]. Third, CYP2D6, the major liver autoantigen in AIH type 2, has been found at the surface of hepatocytes [41]. However, in humans, a definite proof for a pathogenic role of autoantibodies is difficult to come by and would have to involve either a depletion of antibody producing B cells and/or a transfer of disease as a result of a blood transfusion. The initial study with an anti-CD20 antibody (rituximab) therapy that resulted in reduced inflammation in AIH patients [37] might indicate a complicity of autoantibodies. However, it has been shown that the administration of rituximab also affects the CD20+/CD3+ T cell population [42] and thus the effect might not be restricted to B cells.

A major indication for cellular components as drivers of the hepatic damage originates from the composition of immune cell infiltration in the typical pattern of interface hepatitis seen in AIH patients. Lymphocytes, plasma cells, as well as macrophages and monocytes are abundantly present in the cellular infiltrations in the portal tract stretching into the parenchyma [43]. Already in the late 1970s, Vergani et al. demonstrated that lymphocytes of the non-T cell compartment display cytotoxic activity to autologous hepatocytes in HBsAg-negative chronic active hepatitis [44]. But it was not until 1990 that data demonstrating liver autoantigen-specific cytotoxicity of T cells were published by Wen et al. who performed clonal analysis of T cells obtained from the peripheral blood and the liver of AIH patients [45]. From the seven isolated T cell clones, six reacted with the liver-specific membrane lipoprotein (LSP) and one with the asialoglycoprotein receptor (ASGPR). Interestingly, in the blood, the dominant liver autoantigen-specific T cell population had a CD4+ α/β T cell phenotype, whereas in the liver, mainly either CD8+ α/β T cells or CD4/CD8 γ/δ T cells have been found [45]. These early data indicated that cytotoxic T cells are indeed found in the liver of AIH patients and that T cell help might be important for the activation of cytotoxic T cells and the generation of IgG-type autoantibodies. Since its identification as the major target of LKM-1 antibodies in AIH type 2 in the late 1980s [29], CYP2D6 is possibly the one liver autoantigen that has been most thoroughly investigated. Epitope mapping has been conducted for both antibodies [24,25,46] and T cells [14]. Thus, it comes as no surprise that a lot of information on the phenotype and activity of CYP2D6-specific T cell populations has been collected. Lohr et al. isolated 189 T cell clones from liver biopsies of AIH type 2 patients (LKM-1 positive) and found that the majority of liver-infiltrating CYP2D6-specific T cells were CD4+ and proliferated upon stimulation with CYP2D6 [32]. However, they also found cytotoxic activity in 24 out of 26 CD8+ and 20 out of 63 CD4+ T cell clones [32]. A detailed epitope mapping performed with overlapping peptides covering the entire CYP2D6 protein on CYP2D6-specific CD4 T cell clones revealed seven major epitopes [14]. Importantly, Ma et al. demonstrated that the production of the T cell cytokines interferon γ (IFNγ) and interleukin (IL)-4/IL-10 was dependent on the stimulation with distinct peptides, indicating a differential activation pattern of T cells of the T helper (Th) 1-type (IFNγ-producing) and the Th2-type (IL-4/IL-10 producing) during the disease pathogenesis [14]. Epitope spreading, as demonstrated in a mouse model for AIH (see Section 4) [47], might have been involved in such a differential epitope specificity. Th17-type T cells have also been detected in patients with AIH, which supports findings of elevated serum levels of IL-17 and IL-23 as well as increased hepatic expression of IL-17, IL-23, RAR-related orphan receptor γt (RORγt), IL-6, and IL-1β in AIH patients [48]. Interestingly, IL-17 induced a mitogen-activated protein kinase (MAPK)-dependent expression of IL-6 in hepatocytes, which in turn stimulated Th17-type T cells [48]. These data suggest that Th17-type T cells also play an important role in AIH pathogenesis, particularly since it has been shown that transforminf groth factor β (TGFβ) in combination with IL-6 converts CD4 precursor T cells in Th17-type T cells rather than regulatory T cells (Treg) [49]. Indeed, CD4+ CD25^high^ Treg are found to be reduced in numbers and decreased in their suppressive/regulatory activity in patients with AIH [50]. Similarly, it has been demonstrated that CD4+ CD25^high^ Treg from AIH patients are impaired in their ability to suppress IFNγ production by CD4 and CD8 T cells [51]. However, more recently, a study by Peiseler et al. further restricted the analysis to CD4+ CD25+ forkhead box P3 (FoxP3)+ Treg to exclude recently activated CD4 T cells that also express CD25 [52]. They found no impairment of FoxP3+ Treg in AIH patients and an elevated intrahepatic Treg frequency compared to patients with non-alcoholic steatohepatitis (NASH) [52].

The central question is of course how such liver autoantigen-specific B and T cells of any given phenotype have been elicited in the first place. Besides lymphocytes, dominant cell types found in the cellular infiltration of the portal/periportal area include monocytes and macrophages [43,51]. Such monocytes are clearly the result of an inflammatory event, since they predominantly express the pro-inflammatory cytokine TNFα rather than IL-10 [51]. External triggers for a pro-inflammatory milieu in the liver can be manifold, including pathogen infections, drugs, alcohol, and obesity. It has been reported that 26 of 54 patients with NASH also carried ANA or anti-mitochondrial antibodies (AMA) and manifested histological signs of an overlap with AIH or PBC, respectively [53]. In a larger study, it was found that 23% of 225 patients with non-alcoholic fatty liver disease (NAFLD) carried ANA or SMA and the majority (88%) of such autoantibody-positive NAFLD patients fulfilled the diagnostic criteria for AIH [54]. However, in another study with 212 patients with biopsy-proven NAFLD, only one patient could be classified as definite AIH after liver biopsy, although 33% of these patients were ANA-positive [55]. Thus, similar to alcoholic liver disease and its influence on autoimmunity [56], there is neither firm proof that NAFLD or NASH induce or at least promote autoimmune liver disease nor that they influence the severity of disease. However, it has been demonstrated that pre-existing NAFLD leads to an increased frequency of liver autoantigen-specific T cells and a higher severity of AIH in the mouse [57]. Drugs are known to induce acute and/or chronic liver injury and may cause autoimmune-mediated hepatitis with similar manifestations as AIH. However, due to the often known etiology, drug-induced autoimmune hepatitis, such as halothane hepatitis [58], are classified separately from AIH [59]. Yet, there is currently no clear evidence for drugs or xenobiotics to break tolerance to AIH-related liver autoantigens. This stands in contrast to PBC, for which 2-octynoic acid (2-OA), a cosmetic and food additive, has been suggested as a trigger of a liver autoantigen-specific immune response [60]. Patients with PBC carry autoantibodies directed against the E2-subunit of the pyruvate dehydrogenase complex (PDH-E2), the major liver autoantigen in PBC, that cross-react with 2-OA [60]. In addition, immunization of mice with 2-OA conjugated to a carrier protein induces a PBC-like disease [61]. However, such cases of “molecular mimicry” between trigger and target are most often associated with pathogen infection [62,63].

In the context of AIH, predominantly virus infections have been associated with the initiation and/or propagation of the disease [64]. Interestingly, the occurrence of LKM-1 antibodies in chronically hepatitis C virus (HCV)-infected patients might be explained by molecular mimicry, since antibodies binding to the HCV proteins nonstructural protein (NS) 3 and NS5a recognize a specific conformational epitope on CYP2D6 between aa 254 and 288 [65]. In addition, LKM-1 antibodies of AIH type 2 patients recognizing CYP2D6 aa 193–212 have been demonstrated to cross-react with epitopes of HCV (RNA-dependent DNA polymerase NS5 aa 2977–2996) and cytomegalovirus (CMV) (alkaline exonuclease aa 121–140) [66]. A further sequence homology has been reported between the immunodominant CYP2D6 epitope, DPAQPPRD, and the infected cell protein 4 (ICP4) of herpes simplex virus 1 (HSV-1) [31]. Furthermore, we have found additional sequence homologies between a variety of CYP2D6 epitopes and proteins of human pathogens, including *Legionella pneumophila*, influenza A (H1N1) virus, Kaposi’s sarcoma-associated herpes virus (HHV-8), and human cytomegalovirus (HHV-5) [46]. However, definite proof for viruses to directly induce AIH has been elusive to date. Yet it is likely that more than just one environmental trigger is required for the precipitation of an autoimmune disease such as AIH [67].

Pathogen infections usually cause the expression of a broad variety of inflammatory factors, such as cytokines and chemokines. In a comparative study of the serum levels of 21 different cytokines and chemokines in 148 patients with either AIH, PBC or PSC, 54 patients with chronic hepatitis C infection and 50 healthy controls revealed an increased expression of IL-6, IL-8, IL-10, chemokine (C-C motif) ligand 4 (CCL4), CCL26, chemokine (C-X-C motif) ligand (CXCL9), and CXCL10 in all patients with autoimmune liver diseases. In addition, patients with AIH and PBC showed elevated levels of IFNγ, IL-1β, IL-4, IL-5, IL-12p70 and IL-13. Further, a significant increase in IL-2 and IL-15 was detected only in the serum of AIH patients [68]. Possibly due to their role in other autoimmune diseases such as type 1 diabetes (T1D) [69], the chemokines CXCL9 and CXCL10 have been getting more attention than other chemokines in the past. However, CXCL10 has not only been found to be elevated in the serum of patients with AIH [68,70], but also in hepatocytes of AIH patients [71], and was, like CXCL9, associated with disease severity [72,73]. Since CXCR3, the receptor for CXCL9 and CXCL10, is predominantly expressed on activated Th1-type and Th17-type T cells, CXCR3 ligands are central in the attraction of aggressive T cells to the site of inflammation [74].

**Figure 1:**
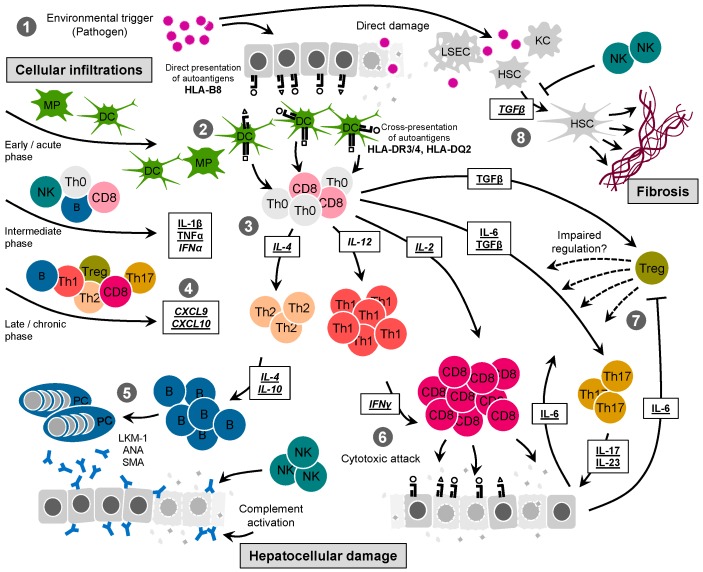
Putative immunopathogenesis of autoimmune hepatitis (AIH). This figure highlights the putative events involved in the immunopathogenesis of AIH as learned from clinical observations and experimental evaluations in animal models. Important inflammatory factors are marked according to whether the evidence was predominantly collected from clinical studies (underlined) or from animal models (italicized). Three main features of AIH are integrated, namely cellular infiltration, hepatocellular damage and hepatic fibrosis. (**1**) One or more environmental triggering events (for example, a liver-tropic pathogen infection) induces direct damage to hepatocytes and possibly also to other liver cells, such as liver-sinusoidal endothelial cells (LSEC), hepatic stellate cells (HSC), and Kupffer cells (KC); (**2**) Liver autoantigens are either directly presented by hepatocytes (mainly by major histocompatibility complex (MHC) class I molecules) or cross-presented by professional antigen-presenting cells, such as dendritic cells (DC) and macrophages (MP), (mainly by MHC class II molecules). Thereby, AIH is associated with the presence of HLA-B8, HLA-DR3, HLA-DR4, and HLA-DQ2. In parallel, the local acute release of alarm signals, such as monocyte-derived cytokines, are released resulting in the attraction and activation of Th0-type T cells, B cells, and natural killer (NK) cells; (**3**) The local pro-inflammatory milieu induces the differentiation of Th0-type T cells predominantly into Th1-type (by interleukin (IL)-12) and Th-17-type (by IL-6 and TGFβ) T cells. However, due to the expression of IL-4, some T cells also acquire a Th2-type phenotype; (**4**) During this phase, the elevated expression of chemokines, such as chemokine (C-X-C motif) ligand (CXCL9) and CXCL10, leads to the attraction of a larger number of immune cells including liver autoantigen-specific T cells as well as non-specific bystander T cells from the circulation to the liver; (**5**) Hepatocyte damage may be precipitated by specific antibodies released by plasma cells (PC), such as liver microsomal antibodies type 1 (LKM-1), anti-nuclear antibodies (ANA), and anti-smooth muscle actin (SMA), that decorate hepatocytes expressing autoantigens at the surface and lead to complement activation and NK cell-mediated killing. Thereby, Th2-type T cells provide help via IL-4 and IL-10 expression; (**6**) Predominant hepatocyte damage seems to originate from cluster of differentiation (CD)8 T cell-mediated killing via perforin/granzyme B-mediated cell lysis or by the release of toxins, such as tumor necrosis factor α (TNFα) and interferon γ (IFNγ); (**7**) Th17-type T cells, via IL-17 and IL-23 release, induce the production of IL-6 by the hepatocytes, which further stimulate Th17-type T cell generation, blocking regulatory T cells (Treg). An impaired immune regulation by Treg is, however, controversial; (**8**) Besides being an important differentiation factor for Th17-type T cells and Treg, transforming growth factor β (TGFβ) is also required for an activation of HSC and subsequently for the exacerbated deposition of collagen observed in hepatic fibrosis. NK cells have been shown to block HSC activation.

In summary, the knowledge gained on the pathogenic mechanism of AIH from studies with sera and biopsies of AIH patients is naturally limited to analytical and observational investigations. Nevertheless, the following possible scenario can be envisioned (Figure 1): An external triggering event, such as a virus infection, elicits an inflammation locally in the liver. Liver resident cells of the innate immune system (macrophages, dendritic cells, neutrophils, natural killer (NK) cells) are activated and release a first wave of inflammatory factors (cytokines, chemokines) that in turn attract and/or activate additional cells of the innate as well as the adaptive immune system, including B and T cells. Maybe due to the existence of a structural similarity between one or more viral antigens and liver autoantigens (molecular mimicry) or by simple presentation of sequestered “unknown” autoantigens upon hepatocyte damage, specific B and T cells are activated and expanded. Subsequently, by mechanisms of hepatocyte lysis (perforin/granzyme B or complement mediated) or release of cellular toxins, such as IFNγ and TNFα, the chronic destruction is perpetuated. Although such a scenario seems feasible and fits well into the canonical events that are described for many other autoimmune diseases, firm proof cannot be gained by observations in patients alone. Thus, from the beginning of the recognition of AIH as an autoimmune-mediated disease, studies have tried to establish animal models to better investigate the mechanism of the disease and evaluate possible therapies.

## 4. Mechanistic Insight from Animal Models

This review does intend to provide a comprehensive listing of all animal models for AIH that have been developed in the past. Its goal is to highlight the knowledge of immunopathogenic mechanisms involved in acute and chronic AIH-like disease and the associated hepatic fibrosis. However, for a more detailed description of the models themselves, we would like to refer to other recent reviews on that topic [75,76]. Many animal models for AIH have been developed over the last three to four decades starting in the early 1970s with the injection of liver homogenates of heterologous origin combined with various activating adjuvants into mice and rabbits by Meyer zu Büschenfelde et al. [77]. The results were promising since they found prolonged flares of hepatitis. Interestingly, it already became clear at that time that the generated antibodies against unknown liver autoantigens were not sufficient to induce disease in transfer experiments, suggesting the additional requirement of pathogenic T cells. In the 1980s, Kuriki et al. developed a mouse model termed “experimental autoimmune hepatitis” or “EAH” by injecting mice with syngeneic liver homogenate or liver-specific lipoproteins with the polysaccharide of *Klebsiella pneumoniae* 03:K1 as an adjuvant [78]. EAH mice generated liver-specific lipoprotein antibodies, had portal infiltrations of mononuclear cells and the transfer of splenocytes from these mice into non-treated recipients resulted in portal infiltration with mononuclear cells and necrosis of parenchymal cells [78]. The presence of liver autoantigen-specific T cells was demonstrated later by Lohse et al., who induced EAH by immunization of C57BL/6 mice with a crude 100,000 g supernatant of syngeneic liver homogenate (S-100) emulsified in complete Freund’s adjuvant [79]. Many follow-up studies used the S-100 injection model in various inbred mouse strains giving rise to valuable information on effector cells and regulatory phenomena in liver-specific immune reactions [80,81,82]. In addition, it has also become evident that an external trigger is required to induce acute and/or chronic hepatitis. In contrast to other autoimmune diseases, such as T1D or PSC, there are no spontaneous models that would serve as a “gold standard” model for the disease, similar to the non-obese diabetic (NOD) mouse for T1D [83] or the multidrug resistance2 (mdr2)−/− mouse for PSC [84]. Nevertheless, some knock-out models of AIH have been established that give insight into the pathogenesis of the disease. For example, programmed cell death 1 (*PD-1*)-deficient mice when neonatally thymectomized (NTx) develop fatal AIH due to a lack of appropriate immune regulation [85]. Similar to regular *PD-1*-deficient mice, *NTxPD-1*-deficient mice display a reduced number of regulatory T cells. However, in contrast to regular *NT*- and untreated *PD-1*-deficient mice, they produced ANA and developed a fatal hepatitis characterized by CD4 and CD8 T cell infiltrations of the liver parenchyma with massive lobular necrosis [85]. Maruoka et al. demonstrated that the administration of dexamethasone to *NTxPD-1-*deficient mice prevented the development of fatal AIH, but not the production of aggressive T cells. Thus, removal of dexamethasone led to a relapse of AIH [86]. Further, it has been shown that blockade of CXCL9 or IL-18 suppressed AIH progression in *NTxPD-1-*deficient mice by decreasing the frequency of CXCR3+ T cells in the liver [73]. Another example for spontaneous AIH-like disease is a triple knock-out mouse model for the receptor tyrosine kinases Tyro3, Axl and Mer that play an important role as negative regulators of Toll-like receptor (TLR) signaling [87]. The excessive stimulation via the TLR signaling pathway in such mice induced persistently elevated serum aminotransferase levels, severe portal inflammation and piecemeal necrosis, as well as presence of ANA and SMA [87].

In contrast to such spontaneous models that develop AIH-like disease due to a naturally occurring or transgenically introduced defect, most model systems use external inflammatory triggers to mimic acute and/or chronic features of human AIH. One of the most frequently used ways to induce cytokine-mediated liver injury in mouse models is the systemic application of the lectin concanavalin A (ConA) that leads to unspecific T cell stimulation and severe liver injury [88]. TNFα and IFNγ have been identified as the final effector cytokines required for ConA-induced hepatitis, since neutralizing antibodies to either cytokine can inhibit the disease [89,90]. It has been further found that the cytokine production depends on the interaction between lymphocytes and macrophages [91] and that Fas ligand (FasL) expression on liver natural killer T cells (NKT) plays a role in the pathogenesis of this model for acute hepatitis [92].

In the 1990s, many models were generated in which disease-related or -unrelated model autoantigens were transgenically expressed as specific targets in the liver. As expected, such animals did not spontaneously develop hepatitis and required the activation of a target-specific immune response by either a strong inflammatory event locally in the liver, such as infection with a liver-tropic pathogen, or the additional liver-specific expression of (a) inflammatory mediator(s) [93,94,95,96,97,98,99,100,101,102,103]. Furthermore, some of these transgenic models required the transfer of target antigen-specific T cells to circumvent the robust regulatory mechanisms of the liver. Especially at the beginning, the results obtained with these various animal models led to the identification of mechanisms involved in the establishment and/or maintenance of tolerance to liver autoantigens and indicated that the generation of a model that accurately reflects the chronic nature of human AIH was not an easy task at all. Yet, some models already led to the identification of key inflammatory factors such as IFNγ. In a model developed by Frank Chisari and colleagues, transgenic mice expressing the hepatitis B virus surface antigen (HBsAg) under the control of the mouse albumin promoter developed a transient form of hepatic injury upon adoptive transfer of activated T cells from HBsAg-primed donor mice [93,94]. The HBsAg-specific immune response was dominated by HBsAg-specific cytotoxic T-lymphocytes (CTL) that triggered apoptosis of hepatocytes expressing HBsAg and released IFNγ upon antigen encounter [93]. Limmer et al. demonstrated that tolerance to liver antigens could only be broken after additional transfer of cells expressing the target antigen and the pro-inflammatory cytokine IL-2 [96]. Alternatively, a parallel infection with a liver-specific pathogen (i.e., *Listeria monocytogenes*) provided a local inflammatory milieu sufficient to break tolerance [96].

Many models followed an adoptive transfer strategy of target antigen-specific TCR-transgenic T cells to circumvent thymic selection of T cells specific to endogenously or transgenically expressed target antigens in the liver. However, due to the lack of an inflammation in the liver, such transfer models rarely induced AIH-like disease. Thus, T cell transfer often was combined with an infection with liver-tropic viruses, bacteria or parasites. Pathogen infection targeting the liver can cause a local “fertile field” [104], which then enhances the attraction of potential aggressive cells of the immune system. Such a strategy was used in transgenic mice expressing the immunodominant CTL epitope GP33 of the glycoprotein (GP) of the lymphocytic choriomeningitis virus (LCMV) under the control of the albumin promoter (Alb) in the liver [97]. This model demonstrated that infection with a liver-tropic pathogen (i.e., LCMV) as well as a the presence of liver autoantigen-specific T cells were required to induce a transient form of hepatitis, since transfer of target antigen-specific T cells or LCMV-infection alone did not induce disease [97]. In addition, the model underlines the perception of the liver as an organ with a high level of immune regulatory activity, since the findings are in contrast to the observation made in the RIP-LCMV model for T1D. In transgenic RIP-LCMV mice, GP is expressed under the control of the rat insulin promoter (RIP) in the insulin-producing β-cells of the pancreas [105,106]. To induce diabetes in RIP-LCMV mice, only infection with LCMV is required, and not a transfer of target antigen-specific T cells [105,107,108]. These data suggest that in contrast to similar models, which have been used to mimic autoimmune diseases affecting other organs, the expression of target antigens on hepatocytes requires a stronger trigger to initiate an autoimmune process. The reason for such a requirement is most likely the immunoprivileged status of the liver. The mechanisms of hepatic immune tolerance include T cell inactivation by antigenic priming in the liver [109], tolerance induction via cross-presentation by liver sinusoidal endothelial cells (LSEC) [110], induction of regulatory T cells [111,112], hepatic stellate cell (HSC)-induced T cell apoptosis [113] and hepatic microenvironment-induced differentiation of regulatory dendritic cells [114]. The first conclusive experiments proving that a neoantigen expressed solely on hepatocytes could lead to T cell tolerance by deletion, anergy, and receptor downregulation came from Ferber et al. [95]. They generated transgenic mice that expressed the MHC class I alloantigen H-2Kb under the inducible carbon-reactive protein (CRP) promoter (CRP-Kb mice) and found that even low levels of H-2Kb expression induced tolerance to allogeneic skin grafts in CRP-Kb mice. This tolerance induced in the liver was strong enough to withstand crossing with mice transgenically expressing a H-2Kb-reactive T cell receptor (TCR) on most of their CD8 T cells [95]. Similarly, the liver-specific ectopic expression of myelin basic protein (MBP), a major oligodendrocyte autoantigen in multiple sclerosis (MS), resulted in the protection from neuroinflammatory disease in a mouse model for MS [112]. Luth et al. demonstrated that the ectopic expression of MBP in the liver, but not in the skin, induced the generation of MBP-specific Treg that blocked the proliferation of MBP-specific effector T cells [112]. The finding that tolerogenic properties of the liver are at least partially responsible for the difficulties in generating a valid model for AIH are supported by another TCR-transgenic model that has been developed by Zierden et al. They found a spontaneous chronic liver inflammation in Alb-HA/CL4-TcR double transgenic mice that express the influenza virus hemagglutinin (HA) under the control of the albumin promoter in the liver as well as HA-specific TCR on the majority of CD8 T cells [103]. Here, an infection with a liver-tropic pathogen was not required to elicit persistent hepatitis, suggesting that the presence of a large quantity of liver antigen-specific CD8 T cells is sufficient to provoke chronic liver inflammation [103]. This model nicely demonstrates how the immunosuppressive mechanisms of the liver may influence the course of disease, since the majority of infiltrating HA-specific CD8 T cells displayed impaired proliferative capabilities and only little effector function. In addition, the liver infiltrates contained a considerable number of regulatory CD4 T cells that suppressed the activation of HA-specific CD8 T cells and thereby might have prevented a full-blown autoimmune destruction of the liver parenchyma [103]. The requirement for T cells for AIH to develop is further supported by data obtained from the Traf6ΔTEC model, in which the expression of the ubiquitin ligase Traf6 was ablated specifically in medullary thymic epithelial cells (mTEC), resulting in a disturbed central tolerance mechanism [115]. Such mice showed features of AIH including interface hepatitis, plasmacytosis, and the generation of liver autoantigen-specific antibodies (ANA and anti-soluble liver antigen (SLA)). Further, Alexandropoulos et al. demonstrated that autoreactive CD4 T cells home to the liver, get activated through presentation of liver autoantigens, and differentiate into Th1-type or Th2-type T cells releasing IFNγ or IL-4, respectively. Subsequently, IFNγ activates cytotoxic CD8 T cells and IL-4 induces the differentiation of plasma cells and the production of autoantibodies [115]. Mice with a defect in the autoimmune-regulator (*AIRE*) gene also possess a defective central tolerance and develop a murine-autoimmune polyendocrine syndrome type 1 (APS-1). Among other autoimmune manifestations, such *AIRE*-deficient mice suffer from AIH and develop autoantibodies to a variety of liver autoantigens [116]. *AIRE*-deficient mice also displayed an elevated intrahepatic Treg frequency, which however was not sufficient to control AIH. In contrast, transfer of polyspecific Treg could ameliorate AIH [116].

Besides the requirements of T cells and a local inflammation for AIH to develop, animal models also provided evidence for an important role of cytokines. In situ presence/expression of cytokines can be achieved, for example, by direct intraperitoneal injection or DNA vaccination. Djilali-Saiah et al. used IL-12 to elicit hepatic damage in TTR-LCMV mice, which express the well-characterized nucleoprotein (NP) of LCMV as a target antigen under the control of the transthyretin (TTR) promoter specifically in the liver [99]. DNA vaccination with plasmids encoding the NP and IL-12 genes resulted in liver damage characterized by cellular infiltration and elevated serum aminotransferase levels. In addition, the generation of a NP-specific CTL response was detected after two months and persisted for up to five months post-vaccination [99]. The importance of IL-12 as a driver of the immunopathogenic process was further demonstrated in an interesting approach by Tamaki et al. They used dendritic cells loaded with well-differentiated hepatocellular carcinoma cells (DC/Hepa1-6) for vaccination of wild-type C57BL/6 mice followed by intraperitoneal injection of recombinant human IL-12 and found a liver-specific inflammation and liver autoantigen-specific proliferative and cytotoxic responses [101]. Interestingly, adoptive transfer of DC/Hepa1-6 activated splenocytes into non-vaccinated recipients resulted in liver damage. However, the recipient mice had to be treated with IL-12 to develop an autoimmune-like hepatitis [101].

## 5. Insight from the CYP2D6 Mouse Model

Some of the models discussed above did not use a defined target liver autoantigen, which makes a quantification and analysis of the specific T cell response difficult. Other strategies employed target proteins that have been well characterized in models for other autoimmune diseases, such as the RIP-LCMV model for T1D [105,108], but had no association with human autoimmune liver disease. In contrast, the CYP2D6 mouse model utilizes the immunodominant antigen (human CYP2D6) in human AIH type 2 [28,29], as a triggering antigen. The targeted delivery of CYP2D6 to the liver with an adenovirus (Ad-2D6) ensures at first an acute hepatic inflammation that subsequently develops into a chronic AIH-like disease [117,118]. Infection of mice with Ad-2D6 results in interface hepatitis with cellular infiltrates that predominantly exhibit B cells and CD4 T cells. However, CD8 T cells, macrophages, dendritic cells, and neutrophils are also frequent [118]. T cell epitope mapping revealed the presence of one CD4 and three distinct CD8 T cell epitopes [47]. Interestingly, these epitopes are all located in regions of intermediate homology between the triggering human CYP2D6 and the target mouse cytochrome P450 (Cyp) homologs, indicating that molecular mimicry rather than identity was required to break Cyp epitope-specific tolerance [47]. This finding was supported by data from human CYP2D6 transgenic mice, which mount almost no T cell immune response [47] and develop a much milder form of AIH [118]. These data indicate that liver autoantigen-specific T cells are one of the main drivers of the pathogenesis of AIH in the mouse. In addition, it serves as proof of principle that an infection with a pathogen that confers molecular mimicry to a liver autoantigen is indeed able to elicit a liver-specific autoimmune disease. However, it has to be stressed here that an infection with a liver-tropic virus was needed to generate a fertile field for the chronic autoimmune destruction to be initiated and/or propagated, since subcutaneous injection of recombinant CYP2D6 and complete Freund’s adjuvant also results in the generation of CYP2D6-specific B and T cells but was not sufficient to cause liver damage and AIH [47]. 

In order to find pathogens that might play a role in the etiology of AIH, the CYP2D6 model was also used to investigate early immunogenic B cell epitopes on the CYP2D6 molecule. Many CYP2D6 epitopes recognized by sera of patients with AIH have been found in the past (reviewed in [25]) and many of these epitopes share sequence homologies to human pathogens [46,119,120]. Since blood samples of AIH patients are mostly only available at or after diagnosis immunoreactivity to the initial immunogenic regions of CYP2D6 might have vanished, leaving behind reactivity to only the most potent immunodominant epitopes. Thus the inducible CYP2D6 model was used to perform a B cell epitope mapping at several defined times after initiation of the disease (i.e., Ad-2D6 infection) in order to reveal to which CYP2D6 epitope antibodies are generated first. Interestingly, the immunodominant CYP2D6 epitope aa 259–270 was also the first epitope to be recognized [46]. This immunodominant epitope contains a core sequence that has also been recognized by sera of the majority of AIH patients [30,31,120,121] and displays sequence homology to the ICP4 of HSV-1 [31]. Unfortunately, there are no firm data from studies with AIH patients that would support the hypothesis that HSV-1 infection plays a role in the etiology of AIH. However, it is noteworthy that reactivity to ICP4 of HSV-1 was frequent in a cohort of 46 patients with chronic hepatitis C including 23 patients with LKM-1 antibodies, but ICP4 reactivity was independent of the presence of LKM-1 antibodies. Further, antibody inhibition studies revealed a true cross-reactivity in only 2 out of 23 LKM-1 antibody-positive hepatitis C patients [119].

The T cell immune response in the CYP2D6 model is dominated by IFNγ-releasing Th1-type T cells [47], whereas Th2-type and Th-17-type T cells secreting IL-4/IL-10 and IL-17, respectively, are far less frequent (Christen and Hintermann, unpublished observation). An additional screening for chemokine expression after Ad-2D6 infection with an inflammatory protein array revealed the induction and/or upregulation of several chemokines including, CCL3, CCL5, CXCL9, and CXCL10 locally in the liver [122]. A similar upregulation of chemokine expression has been reported by Milks et al. who assessed the global gene expression profile by microarray analysis of mRNA isolated from the livers of *Tgfb1*-deficient Balb/c mice to develop a fulminant AIH. They found the highest upregulation (>10-fold) for the chemokines CCL3, CCL4, CCL5, CCL6, CCL24, CXCL2, CXCL9, CXCL10 and CXCL11 [123]. In the CYP2D6 model, a closer look at CXCL10 and CCL5 using the according knock-out deficient mouse lines revealed that CXCL10 and CCL5 might have opposite effects on the pathogenesis of AIH. Whereas in absence of CXCL10 the infiltration of the liver was reduced, the absence of CCL5 resulted in an exacerbation of the disease as documented by an enhanced infiltration of the liver and an elevation of the frequency of activated CD4 T cells [122]. These data confirm findings in AIH patients indicating that CXCL10 might be one of the main drivers of the disease. In contrast, it has been demonstrated in the ConA model for acute immune-mediated hepatitis that a lack of CXCR3 exacerbates liver damage and abolishes tolerance upon ConA restimulation [124]. This seems to stand in contradiction to the findings in patients and the CYP2D6 model, but one has to consider that CXCL9 as well as CXCL10 are binding to CXCR3 and therefore the biological activity of both ligands are neutralized in *CXCR3*-deficient mice, whereas in *CXCL10*-deficient mice CXCL9–CXCR3 interaction is still possible. It has been suggested that the CXCL9 and CXCL10 might have opposite effects due to a differential binding to the receptor [74,125].

One of the advantages of the CYP2D6 model over other animal models for AIH is the development of chronic liver fibrosis. Infection with Ad-2D6 induces a chronic activation of HSC resulting in an increased deposition of extracellular matrix, such as collagen I and III, and elevated expression of α-smooth muscle actin (αSMA) predominantly in and underneath the capsule [126]. Therefore the route of Ad-2D6 injection was important for fibrosis development, since intravenous administration resulted in a disproportional recruitment of NK cells preventing an efficient chronic HSC activation and fibrosis. In contrast, intraperitoneal administration resulted in a much lower frequency of NK cell in the liver and caused pronounced subcapsular fibrosis and clustering of inflammatory monocytes [126].

## 6. Conclusions and Future Perspectives

Reviews on autoimmune hepatitis often contain statements that not much is known about the etiology of the disease and that the mechanisms of the hepatic damage have not been uncovered in detail. Such statements are, on the one hand, true—firm proof for initiating events, such as virus infections, are still lacking and the immunopathogenic path of hepatic destruction still contains large gaps of understanding. On the other hand, one has to acknowledge the progress that has been made in the past three decades from the discovery of chronic active hepatitis that was often associated with a dire long-term prognosis and the requirement of a liver transplant, to current therapeutic interventions that result in a favorable outcome for most patients. Nevertheless, the traditional treatment regimen is still heavily dependent on the administration of corticosteroids and cytostatic drugs that often are associated with undesired side effects. Thus, it is important to effectively transfer the knowledge gained from studies with AIH patients and from animal models to the development of novel therapies. As described in this review and depicted in Figure 1, many immunopathogenic mechanisms have been identified in AIH patients as well as in animal models. The putative chain of events that are involved in the immunopathogenesis of AIH as depicted in Figure 1 offers a broad variety of specific intervention sites. Hence, animal models are a perfect testing ground to evaluate innovative treatments, such as the neutralization of critical inflammatory factors or the transfer of liver autoantigen-specific Treg. In this context, it is important to stress that none of the current animals represents human AIH with all its features. In particular, the immunoreactivity to human liver autoantigens is, with the exception of the AIH type 2 autoantigens CYP2D6 and formiminotransferase cyclodeaminase (FTCD) [127], not covered by any of the current models. Since the majority of AIH patients carry antibodies and T cells directed against other liver autoantigens there is still need for better models to be used as an equivalent to human AIH, particularly for AIH type 1.

## Abbreviations

AIH	autoimmune hepatitis
PBC	primary biliary cholangitis
PSC	primary sclerosing cholangitis
ANA	anti-nuclear antibody
SMA	anti-smooth muscle antibody
LKM-1	type 1 liver/kidney microsomal autoantibodies
CYP2D6	cytochrome P450 2D6
LSP	liver-specific membrane lipoprotein
ASGPR	asialoglycoprotein receptor
MAPK	mitogen-activated protein kinase
NASH	non-alcoholic steatohepatitis
NAFLD	non-alcoholic fatty liver disease
PDH-E2	E2-subunit of the pyruvate dehydrogenase complex
2-OA	2-octynoic acid
CMV	cytomegalovirus
ICP4	infected cell protein 4
HSV-1	herpes simplex virus 1
HHV-8	Kaposi’s sarcoma-associated herpes virus
HHV-5	human cytomegalovirus
EAH	experimental autoimmune hepatitis
S-100	100,000 g supernatant of syngeneic liver homogenate
NOD	non-obese diabetic
PD-1	programmed cell death
NT	neonatal thymectomized
TLR	toll-like receptor
ConA	concanavalin A
NKT	natural killer T cells
HBsAg	hepatitis B virus surface antigen
CTL	cytotoxic T-lymphocytes
GP	glycoprotein
LCMV	lymphocytic choriomeningitis virus
Alb	albumin promoter
RIP	rat insulin promoter
LSEC	liver sinusoidal endothelial cells
HSC	hepatic stellate cell
CRP	carbon-reactive protein
TCR	T cell receptor
MBP	myelin basic protein
HA	hemagglutinin
mTEC	medullary thymic epithelial cells
AIRE	autoimmune regulator
APS-1	autoimmune polyendocrine syndrome type 1
TTR	transthyretin
NP	nucleoprotein
Ad-2D6	adenovirus expressing CYP2D6
αSMA	α-smooth muscle actin
FTCD	formiminotransferase cyclodeaminase

## References

[1] Czaja A.J. (2015). Diagnosis and management of autoimmune hepatitis. Clin. Liver Dis..

[2] Liberal R., Grant C.R., Mieli-Vergani G., Vergani D. (2013). Autoimmune hepatitis: A comprehensive review. J. Autoimmun..

[3] Vierling J.M. (2012). Diagnosis and treatment of autoimmune hepatitis. Curr. Gastroenterol. Rep..

[4] Lohse A.W., Wiegard C. (2011). Diagnostic criteria for autoimmune hepatitis. Best Pract. Res. Clin. Gastroenterol..

[5] Manns M.P., Lohse A.W., Vergani D. (2015). Autoimmune hepatitis—Update 2015. J. Hepatol..

[6] Boberg K.M. (2002). Prevalence and epidemiology of autoimmune hepatitis. Clin. Liver Dis..

[7] Czaja A.J. (2006). Autoimmune hepatitis—Approach to diagnosis. MedGenMed.

[8] World Health Organization. Dept. of Protection of the Human Environment, Inter-Organization Programme for the Sound Management of Chemicals (2006). Principles and Methods for Assessing Autoimmunity Associated with Exposure to Chemicals. http://www.who.int/iris/handle/10665/43603.

[9] Manns M.P., Czaja A.J., Gorham J.D., Krawitt E.L., Mieli-Vergani G., Vergani D., Vierling J.M. (2010). Diagnosis and management of autoimmune hepatitis. Hepatology.

[10] Donaldson P.T., Doherty D.G., Hayllar K.M., McFarlane I.G., Johnson P.J., Williams R. (1991). Susceptibility to autoimmune chronic active hepatitis: Human leukocyte antigens DR4 and A1-B8-DR3 are independent risk factors. Hepatology.

[11] Doherty D.G., Donaldson P.T., Underhill J.A., Farrant J.M., Duthie A., Mieli-Vergani G., McFarlane I.G., Johnson P.J., Eddleston A.L., Mowat A.P. (1994). Allelic sequence variation in the HLA class II genes and proteins in patients with autoimmune hepatitis. Hepatology.

[12] Czaja A.J., Carpenter H.A., Santrach P.J., Moore S.B. (1993). Significance of HLA DR4 in type 1 autoimmune hepatitis. Gastroenterology.

[13] Donaldson P.T. (2004). Genetics of liver disease: Immunogenetics and disease pathogenesis. Gut.

[14] Ma Y., Bogdanos D.P., Hussain M.J., Underhill J., Bansal S., Longhi M.S., Cheeseman P., Mieli-Vergani G., Vergani D. (2006). Polyclonal T-cell responses to cytochrome P450IID6 are associated with disease activity in autoimmune hepatitis type 2. Gastroenterology.

[15] Djilali-Saiah I., Renous R., Caillat-Zucman S., Debray D., Alvarez F. (2004). Linkage disequilibrium between HLA class II region and autoimmune hepatitis in pediatric patients. J. Hepatol..

[16] Liberal R., Krawitt E.L., Vierling J.M., Manns M.P., Mieli-Vergani G., Vergani D. (2016). Cutting edge issues in autoimmune hepatitis. J. Autoimmun..

[17] De Boer Y.S., van Gerven N.M., Zwiers A., Verwer B.J., van Hoek B., van Erpecum K.J., Beuers U., van Buuren H.R., Drenth J.P., den Ouden J.W. (2014). Genome-wide association study identifies variants associated with autoimmune hepatitis type 1. Gastroenterology.

[18] Manns M.P., Strassburg C.P. (2001). Autoimmune hepatitis: Clinical challenges. Gastroenterology.

[19] Van Gerven N.M., de Boer Y.S., Zwiers A., Verwer B.J., Drenth J.P., van Hoek B., van Erpecum K.J., Beuers U., van Buuren H.R., den Ouden J.W. (2015). HLA-DRB1*03:01 and HLA-DRB1*04:01 modify the presentation and outcome in autoimmune hepatitis type-1. Genes Immun..

[20] Kirstein M.M., Metzler F., Geiger E., Heinrich E., Hallensleben M., Manns M.P., Vogel A. (2015). Prediction of short- and long-term outcome in patients with autoimmune hepatitis. Hepatology.

[21] Czaja A.J. (2013). Challenges in the diagnosis and management of autoimmune hepatitis. Can. J. Gastroenterol..

[22] Strassburg C.P., Manns M.P. (2011). Therapy of autoimmune hepatitis. Best Pract. Res. Clin. Gastroenterol..

[23] Hennes E.M., Zeniya M., Czaja A.J., Pares A., Dalekos G.N., Krawitt E.L., Bittencourt P.L., Porta G., Boberg K.M., Hofer H. (2008). Simplified criteria for the diagnosis of autoimmune hepatitis. Hepatology.

[24] Liberal R., Mieli-Vergani G., Vergani D. (2013). Clinical significance of autoantibodies in autoimmune hepatitis. J. Autoimmun..

[25] Christen U. (2013). The role of autoantibodies in autoimmune hepatitis type 2. Immunotherapy.

[26] Krawitt E.L. (2006). Autoimmune hepatitis. N. Engl. J. Med..

[27] Muratori P., Lalanne C., Fabbri A., Cassani F., Lenzi M., Muratori L. (2015). Type 1 and type 2 autoimmune hepatitis in adults share the same clinical phenotype. Aliment. Pharmacol. Ther..

[28] Manns M.P., Johnson E.F., Griffin K.J., Tan E.M., Sullivan K.F. (1989). Major antigen of liver kidney microsomal autoantibodies in idiopathic autoimmune hepatitis is cytochrome P450db1. J. Clin. Investig..

[29] Zanger U.M., Hauri H.P., Loeper J., Homberg J.C., Meyer U.A. (1988). Antibodies against human cytochrome P-450db1 in autoimmune hepatitis type II. Proc. Natl. Acad. Sci. USA.

[30] Yamamoto A.M., Cresteil D., Boniface O., Clerc F.F., Alvarez F. (1993). Identification and analysis of cytochrome P450IID6 antigenic sites recognized by anti-liver-kidney microsome type-1 antibodies (LKM1). Eur. J. Immunol..

[31] Manns M.P., Griffin K.J., Sullivan K.F., Johnson E.F. (1991). LKM-1 autoantibodies recognize a short linear sequence in P450IID6, a cytochrome P-450 monooxygenase. J. Clin. Investig..

[32] Lohr H., Manns M., Kyriatsoulis A., Lohse A.W., Trautwein C., Meyer zum Buschenfelde K.H., Fleischer B. (1991). Clonal analysis of liver-infiltrating T cells in patients with LKM-1 antibody-positive autoimmune chronic active hepatitis. Clin. Exp. Immunol..

[33] Lohr H.F., Schlaak J.F., Lohse A.W., Bocher W.O., Arenz M., Gerken G., Meyer Zum Buschenfelde K.H. (1996). Autoreactive CD4+ LKM-specific and anticlonotypic T-cell responses in LKM-1 antibody-positive autoimmune hepatitis. Hepatology.

[34] Czaja A.J. (2012). Advances in the current treatment of autoimmune hepatitis. Dig. Dis. Sci..

[35] Zachou K., Gatselis N., Papadamou G., Rigopoulou E.I., Dalekos G.N. (2011). Mycophenolate for the treatment of autoimmune hepatitis: Prospective assessment of its efficacy and safety for induction and maintenance of remission in a large cohort of treatment-naive patients. J. Hepatol..

[36] Weiler-Normann C., Schramm C., Quaas A., Wiegard C., Glaubke C., Pannicke N., Moller S., Lohse A.W. (2013). Infliximab as a rescue treatment in difficult-to-treat autoimmune hepatitis. J. Hepatol..

[37] Burak K.W., Swain M.G., Santodomingo-Garzon T., Lee S.S., Urbanski S.J., Aspinall A.I., Coffin C.S., Myers R.P. (2013). Rituximab for the treatment of patients with autoimmune hepatitis who are refractory or intolerant to standard therapy. Can. J. Gastroenterol..

[38] Boberg K.M., Chapman R.W., Hirschfield G.M., Lohse A.W., Manns M.P., Schrumpf E. (2011). Overlap syndromes: The International Autoimmune Hepatitis Group (IAIHG) position statement on a controversial issue. J. Hepatol..

[39] Ma Y., Okamoto M., Thomas M.G., Bogdanos D.P., Lopes A.R., Portmann B., Underhill J., Durr R., Mieli-Vergani G., Vergani D. (2002). Antibodies to conformational epitopes of soluble liver antigen define a severe form of autoimmune liver disease. Hepatology.

[40] Vergani D., Mieli-Vergani G., Mondelli M., Portmann B., Eddleston A.L. (1987). Immunoglobulin on the surface of isolated hepatocytes is associated with antibody-dependent cell-mediated cytotoxicity and liver damage. Liver.

[41] Muratori L., Parola M., Ripalti A., Robino G., Muratori P., Bellomo G., Carini R., Lenzi M., Landini M.P., Albano E. (2000). Liver/kidney microsomal antibody type 1 targets CYP2D6 on hepatocyte plasma membrane. Gut.

[42] Schuh E., Berer K., Mulazzani M., Feil K., Meinl I., Lahm H., Krane M., Lange R., Pfannes K., Subklewe M. (2016). Features of Human CD3^+^CD20^+^ T Cells. J. Immunol..

[43] Senaldi G., Portmann B., Mowat A.P., Mieli-Vergani G., Vergani D. (1992). Immunohistochemical features of the portal tract mononuclear cell infiltrate in chronic aggressive hepatitis. Arch. Dis. Child..

[44] Vergani G.M., Vergani D., Jenkins P.J., Portmann B., Mowat A.P., Eddleston A.L., Williams R. (1979). Lymphocyte cytotoxicity to autologous hepatocytes in HBsAg-negative chronic active hepatitis. Clin. Exp. Immunol..

[45] Wen L., Peakman M., Lobo-Yeo A., McFarlane B.M., Mowat A.P., Mieli-Vergani G., Vergani D. (1990). T-cell-directed hepatocyte damage in autoimmune chronic active hepatitis. Lancet.

[46] Hintermann E., Holdener M., Bayer M., Loges S., Pfeilschifter J.M., Granier C., Manns M.P., Christen U. (2011). Epitope spreading of the anti-CYP2D6 antibody response in patients with autoimmune hepatitis and in the CYP2D6 mouse model. J. Autoimmun..

[47] Ehser J., Holdener M., Christen S., Bayer M., Pfeilschifter J.M., Hintermann E., Bogdanos D., Christen U. (2013). Molecular mimicry rather than identity breaks T-cell tolerance in the CYP2D6 mouse model for human autoimmune hepatitis. J. Autoimmun..

[48] Zhao L., Tang Y., You Z., Wang Q., Liang S., Han X., Qiu D., Wei J., Liu Y., Shen L. (2011). Interleukin-17 contributes to the pathogenesis of autoimmune hepatitis through inducing hepatic interleukin-6 expression. PLoS ONE.

[49] Weaver C.T., Hatton R.D. (2009). Interplay between the TH17 and TReg cell lineages: A (co-)evolutionary perspective. Nat. Rev. Immunol..

[50] Longhi M.S., Ma Y., Bogdanos D.P., Cheeseman P., Mieli-Vergani G., Vergani D. (2004). Impairment of CD4+CD25+ regulatory T-cells in autoimmune liver disease. J. Hepatol..

[51] Longhi M.S., Mitry R.R., Samyn M., Scalori A., Hussain M.J., Quaglia A., Mieli-Vergani G., Ma Y., Vergani D. (2009). Vigorous activation of monocytes in juvenile autoimmune liver disease escapes the control of regulatory T-cells. Hepatology.

[52] Peiseler M., Sebode M., Franke B., Wortmann F., Schwinge D., Quaas A., Baron U., Olek S., Wiegard C., Lohse A.W. (2012). FOXP3+ regulatory T cells in autoimmune hepatitis are fully functional and not reduced in frequency. J. Hepatol..

[53] Tsuneyama K., Baba H., Kikuchi K., Nishida T., Nomoto K., Hayashi S., Miwa S., Nakajima T., Nakanishi Y., Masuda S. (2013). Autoimmune features in metabolic liver disease: A single-center experience and review of the literature. Clin. Rev. Allergy Immunol..

[54] Adams L.A., Lindor K.D., Angulo P. (2004). The prevalence of autoantibodies and autoimmune hepatitis in patients with nonalcoholic Fatty liver disease. Am. J. Gastroenterol..

[55] Yatsuji S., Hashimoto E., Kaneda H., Taniai M., Tokushige K., Shiratori K. (2005). Diagnosing autoimmune hepatitis in nonalcoholic fatty liver disease: Is the International Autoimmune Hepatitis Group scoring system useful?. J. Gastroenterol..

[56] Albano E. (2012). Role of adaptive immunity in alcoholic liver disease. Int. J. Hepatol..

[57] Muller P., Messmer M., Bayer M., Pfeilschifter J.M., Hintermann E., Christen U. (2016). Non-alcoholic fatty liver disease (NAFLD) potentiates autoimmune hepatitis in the CYP2D6 mouse model. J. Autoimmun..

[58] Gut J., Christen U., Frey N., Koch V., Stoffler D. (1995). Molecular mimicry in halothane hepatitis: Biochemical and structural characterization of lipoylated autoantigens. Toxicology.

[59] Czaja A.J. (2011). Drug-induced autoimmune-like hepatitis. Dig. Dis. Sci..

[60] Amano K., Leung P.S., Rieger R., Quan C., Wang X., Marik J., Suen Y.F., Kurth M.J., Nantz M.H., Ansari A.A. (2005). Chemical xenobiotics and mitochondrial autoantigens in primary biliary cirrhosis: Identification of antibodies against a common environmental, cosmetic, and food additive, 2-octynoic acid. J. Immunol..

[61] Wakabayashi K., Lian Z.X., Leung P.S., Moritoki Y., Tsuneyama K., Kurth M.J., Lam K.S., Yoshida K., Yang G.X., Hibi T. (2008). Loss of tolerance in C57BL/6 mice to the autoantigen E2 subunit of pyruvate dehydrogenase by a xenobiotic with ensuing biliary ductular disease. Hepatology.

[62] Oldstone M.B.A. (1987). Molecular mimicry and autoimmune disease. Cell.

[63] Christen U., Shoenfeld Y., Meroni P.L., Gershwin M.E. (2014). Molecular mimiry. Autoantibodies.

[64] Christen U., Hintermann E. (2014). Pathogen infection as a possible cause for autoimmune hepatitis. Int. Rev. Immunol..

[65] Marceau G., Lapierre P., Beland K., Soudeyns H., Alvarez F. (2005). LKM1 autoantibodies in chronic hepatitis C infection: A case of molecular mimicry?. Hepatology.

[66] Kerkar N., Choudhuri K., Ma Y., Mahmoud A., Bogdanos D.P., Muratori L., Bianchi F., Williams R., Mieli-Vergani G., Vergani D. (2003). Cytochrome P4502D6_193–212_: A new immunodominant epitope and target of virus/self cross-reactivity in liver kidney microsomal autoantibody type 1-positive liver disease. J. Immunol..

[67] Christen U. (2014). Pathogen infection and autoimmunity. Int. Rev. Immunol..

[68] Landi A., Weismuller T.J., Lankisch T.O., Santer D.M., Tyrrell D.L., Manns M.P., Houghton M. (2014). Differential serum levels of eosinophilic eotaxins in primary sclerosing cholangitis, primary biliary cirrhosis, and autoimmune hepatitis. J. Interferon Cytokine Res..

[69] Antonelli A., Ferrari S.M., Giuggioli D., Ferrannini E., Ferri C., Fallahi P. (2014). Chemokine (C-X-C motif) ligand (CXCL)10 in autoimmune diseases. Autoimmun. Rev..

[70] Czaja A.J. (2014). Current and prospective pharmacotherapy for autoimmune hepatitis. Expert Opin. Pharm..

[71] Nishioji K., Okanoue T., Itoh Y., Narumi S., Sakamoto M., Nakamura H., Morita A., Kashima K. (2001). Increase of chemokine interferon-inducible protein-10 (IP-10) in the serum of patients with autoimmune liver diseases and increase of its mRNA expression in hepatocytes. Clin. Exp. Immunol..

[72] Li Y.L., Liu N., Zhao D.T., Li Z.M., Zhang H.P., Liu Y.M., Yan H.P., Zhao Y. (2013). Investigate circulating levels of chemokines and evaluate the correlation between these chemokines and liver function indicators in autoimmune hepatitis. Zhonghua Gan Zang Bing Za Zhi.

[73] Ikeda A., Aoki N., Kido M., Iwamoto S., Nishiura H., Maruoka R., Chiba T., Watanabe N. (2014). Progression of autoimmune hepatitis is mediated by IL-18-producing dendritic cells and hepatic CXCL9 expression in mice. Hepatology.

[74] Christen U., Parnham M. (2015). CXCR3 and Its Ligands. Encyclopedia of Inflammatory Diseases.

[75] Yuksel M., Laukens D., Heindryckx F., van Vlierberghe H., Geerts A., Wong F.S., Wen L., Colle I. (2014). Hepatitis mouse models: From acute-to-chronic autoimmune hepatitis. Int. J. Exp. Pathol..

[76] Christen U., Hintermann E. (2015). An update on animal models of autoimmune hepatitis: Are we there yet?. Curr. Pharm. Des..

[77] Meyer zum Buschenfelde K.H., Kossling F.K., Miescher P.A. (1972). Experimental chronic active hepatitis in rabbits following immunization with human liver proteins. Clin. Exp. Immunol..

[78] Kuriki J., Murakami H., Kakumu S., Sakamoto N., Yokochi T., Nakashima I., Kato N. (1983). Experimental autoimmune hepatitis in mice after immunization with syngeneic liver proteins together with the polysaccharide of Klebsiella pneumoniae. Gastroenterology.

[79] Lohse A.W., Manns M., Dienes H.P., Meyer zum Buschenfelde K.H., Cohen I.R. (1990). Experimental autoimmune hepatitis: disease induction, time course and T-cell reactivity. Hepatology.

[80] Watanabe Y., Kawakami H., Kawamoto H., Ikemoto Y., Masuda K., Takezaki E., Nakanishi T., Kajiyama G., Takeno H. (1987). Effect of neonatal thymectomy on experimental autoimmune hepatitis in mice. Clin. Exp. Immunol..

[81] Asano M., Toda M., Sakaguchi N., Sakaguchi S. (1996). Autoimmune disease as a consequence of developmental abnormality of a T cell subpopulation. J. Exp. Med..

[82] Lohse A.W., Dienes H.P., Meyer zum Buschenfelde K.H. (1998). Suppression of murine experimental autoimmune hepatitis by T-cell vaccination or immunosuppression. Hepatology.

[83] Makino S., Kunimoto K., Muraoka Y., Mizushima Y., Katagiri K., Tochino Y. (1980). Breeding of a non-obese, diabetic strain of mice. Jikken Dobutsu.

[84] Mauad T.H., van Nieuwkerk C.M., Dingemans K.P., Smit J.J., Schinkel A.H., Notenboom R.G., van den Bergh Weerman M.A., Verkruisen R.P., Groen A.K., Oude Elferink R.P. (1994). Mice with homozygous disruption of the mdr2 P-glycoprotein gene. A novel animal model for studies of nonsuppurative inflammatory cholangitis and hepatocarcinogenesis. Am. J. Pathol..

[85] Kido M., Watanabe N., Okazaki T., Akamatsu T., Tanaka J., Saga K., Nishio A., Honjo T., Chiba T. (2008). Fatal autoimmune hepatitis induced by concurrent loss of naturally arising regulatory T cells and PD-1-mediated signaling. Gastroenterology.

[86] Maruoka R., Aoki N., Kido M., Iwamoto S., Nishiura H., Ikeda A., Chiba T., Watanabe N. (2013). Splenectomy prolongs the effects of corticosteroids in mouse models of autoimmune hepatitis. Gastroenterology.

[87] Qi N., Liu P., Zhang Y., Wu H., Chen Y., Han D. (2013). Development of a spontaneous liver disease resembling autoimmune hepatitis in mice lacking Tyro3, Axl and Mer receptor tyrosine kinases. PLoS ONE.

[88] Tiegs G., Hentschel J., Wendel A. (1992). A T cell-dependent experimental liver injury in mice inducible by concanavalin A.. J. Clin. Investig..

[89] Kusters S., Gantner F., Kunstle G., Tiegs G. (1996). Interferon gamma plays a critical role in T cell-dependent liver injury in mice initiated by concanavalin A. Gastroenterology.

[90] Gantner F., Leist M., Lohse A.W., Germann P.G., Tiegs G. (1995). Concanavalin A-induced T-cell-mediated hepatic injury in mice: The role of tumor necrosis factor. Hepatology.

[91] Gantner F., Leist M., Kusters S., Vogt K., Volk H.D., Tiegs G. (1996). T cell stimulus-induced crosstalk between lymphocytes and liver macrophages results in augmented cytokine release. Exp. Cell Res..

[92] Takeda K., Hayakawa Y., van Kaer L., Matsuda H., Yagita H., Okumura K. (2000). Critical contribution of liver natural killer T cells to a murine model of hepatitis. Proc. Natl. Acad. Sci. USA.

[93] Ando K., Guidotti L.G., Wirth S., Ishikawa T., Missale G., Moriyama T., Schreiber R.D., Schlicht H.J., Huang S.N., Chisari F.V. (1994). Class I-restricted cytotoxic T lymphocytes are directly cytopathic for their target cells in vivo. J. Immunol..

[94] Moriyama T., Guilhot S., Klopchin K., Moss B., Pinkert C.A., Palmiter R.D., Brinster R.L., Kanagawa O., Chisari F.V. (1990). Immunobiology and pathogenesis of hepatocellular injury in hepatitis B virus transgenic mice. Science.

[95] Ferber I., Schoenrich G., Schenkel J., Mellor A.L., Haemmerling G.J., Arnold B. (1994). Levels of peripheral T cell tolerance induced by different doses of tolerogen. Science.

[96] Limmer A., Sacher T., Alferink J., Kretschmar M., Schonrich G., Nichterlein T., Arnold B., Hammerling G.J. (1998). Failure to induce organ-specific autoimmunity by breaking of tolerance: Importance of the microenvironment. Eur. J. Immunol..

[97] Voehringer D., Blaser C., Grawitz A.B., Chisari F.V., Buerki K., Pircher H. (2000). Break of T cell ignorance to a viral antigen in the liver induces hepatitis. J. Immunol..

[98] Sacher T., Knolle P., Nichterlein T., Arnold B., Hammerling G.J., Limmer A. (2002). CpG-ODN-induced inflammation is sufficient to cause T-cell-mediated autoaggression against hepatocytes. Eur. J. Immunol..

[99] Djilali-Saiah I., Lapierre P., Vittozi S., Alvarez F. (2002). DNA vaccination breaks tolerance for a neo-self antigen in liver: A transgenic murine model of autoimmune hepatitis. J. Immunol..

[100] Lapierre P., Djilali-Saiah I., Vitozzi S., Alvarez F. (2004). A murine model of type 2 autoimmune hepatitis: Xenoimmunization with human antigens. Hepatology.

[101] Tamaki S., Homma S., Enomoto Y., Komita H., Zeniya M., Ohno T., Toda G. (2005). Autoimmune hepatic inflammation by vaccination of mice with dendritic cells loaded with well-differentiated hepatocellular carcinoma cells and administration of interleukin-12. Clin. Immunol..

[102] Derkow K., Loddenkemper C., Mintern J., Kruse N., Klugewitz K., Berg T., Wiedenmann B., Ploegh H.L., Schott E. (2007). Differential priming of CD8 and CD4 T-cells in animal models of autoimmune hepatitis and cholangitis. Hepatology.

[103] Zierden M., Kuhnen E., Odenthal M., Dienes H.P. (2010). Effects and regulation of autoreactive CD8+ T cells in a transgenic mouse model of autoimmune hepatitis. Gastroenterology.

[104] Von Herrath M.G., Fujinami R.S., Whitton J.L. (2003). Microorganisms and autoimmunity: Making the barren field fertile. Nat. Rev. Microbiol..

[105] Oldstone M.B.A., Nerenberg M., Southern P., Price J., Lewicki H. (1991). Virus infection triggers insulin-dependent diabetes mellitus in a transgenic model: Role of anti-self (virus) immune response. Cell.

[106] Ohashi P., Oehen S., Buerki K., Pircher H., Ohashi C., Odermatt B., Malissen B., Zinkernagel R., Hengartner H. (1991). Ablation of tolerance and induction of diabetes by virus infection in viral antigen transgenic mice. Cell.

[107] Von Herrath M.G., Dockter J., Oldstone M.B.A. (1994). How virus induces a rapid or slow onset insulin-dependent diabetes mellitus in a transgenic model. Immunity.

[108] Christen U., Bender C., von Herrath M.G. (2012). Infection as a cause of type 1 diabetes?. Curr. Opin. Rheumatol..

[109] Bowen D.G., Zen M., Holz L., Davis T., McCaughan G.W., Bertolino P. (2004). The site of primary T cell activation is a determinant of the balance between intrahepatic tolerance and immunity. J. Clin. Investig..

[110] Thomson A.W., Knolle P.A. (2010). Antigen-presenting cell function in the tolerogenic liver environment. Nat. Rev. Immunol..

[111] Crispe I.N. (2003). Hepatic T cells and liver tolerance. Nat. Rev. Immunol..

[112] Luth S., Huber S., Schramm C., Buch T., Zander S., Stadelmann C., Bruck W., Wraith D.C., Herkel J., Lohse A.W. (2008). Ectopic expression of neural autoantigen in mouse liver suppresses experimental autoimmune neuroinflammation by inducing antigen-specific Tregs. J. Clin. Investig..

[113] Chen C.H., Kuo L.M., Chang Y., Wu W., Goldbach C., Ross M.A., Stolz D.B., Chen L., Fung J.J., Lu L. (2006). In vivo immune modulatory activity of hepatic stellate cells in mice. Hepatology.

[114] Xia S., Guo Z., Xu X., Yi H., Wang Q., Cao X. (2008). Hepatic microenvironment programs hematopoietic progenitor differentiation into regulatory dendritic cells, maintaining liver tolerance. Blood.

[115] Alexandropoulos K., Bonito A.J., Weinstein E.G., Herbin O. (2015). Medullary thymic epithelial cells and central tolerance in autoimmune hepatitis development: Novel perspective from a new mouse model. Int. J. Mol. Sci..

[116] Hardtke-Wolenski M., Taubert R., Noyan F., Sievers M., Dywicki J., Schlue J., Falk C.S., Ardesjo Lundgren B., Scott H.S., Pich A. (2015). Autoimmune hepatitis in a murine autoimmune polyendocrine syndrome type 1 model is directed against multiple autoantigens. Hepatology.

[117] Hintermann E., Ehser J., Christen U. (2012). The CYP2D6 Animal Model: How to Induce Autoimmune Hepatitis in Mice. J. Vis. Exp..

[118] Holdener M., Hintermann E., Bayer M., Rhode A., Rodrigo E., Hintereder G., Johnson E.F., Gonzalez F.J., Pfeilschifter J., Manns M.P. (2008). Breaking tolerance to the natural human liver autoantigen cytochrome P450 2D6 by virus infection. J. Exp. Med..

[119] Bogdanos D.P., Lenzi M., Okamoto M., Rigopoulou E.I., Muratori P., Ma Y., Muratori L., Tsantoulas D., Mieli-Vergani G., Bianchi F.B. (2004). Multiple viral/self immunological cross-reactivity in liver kidney microsomal antibody positive hepatitis C virus infected patients is associated with the possession of HLA B51. Int. J. Immunopathol. Pharmacol..

[120] Gueguen M., Boniface O., Bernard O., Clerc F., Cartwright T., Alvarez F. (1991). Identification of the main epitope on human cytochrome P450 IID6 recognized by anti-liver kidney microsome antibody. J. Autoimmun..

[121] Kitazawa E., Igarashi T., Kawaguchi N., Matsushima H., Kawashima Y., Hankins R.W., Miyakawa H. (2001). Differences in Anti-LKM-1 Autoantibody Immunoreactivity to CYP2D6 Antigenic Sites Between Hepatitis C Virus-negative and -positive Patients. J. Autoimmun..

[122] Ehser J., Müller P., Bayer M., Pfeilschifter J.M., Hintermann E., Christen U. (2016). Absence of CXCL10 or CCL5 has opposite effects on the severity of autoimmune hepatitis in the CYP2D6 mouse model.

[123] Milks M.W., Cripps J.G., Lin H., Wang J., Robinson R.T., Sargent J.L., Whitfield M.L., Gorham J.D. (2009). The role of *Ifng* in alterations in liver gene expression in a mouse model of fulminant autoimmune hepatitis. Liver Int..

[124] Erhardt A., Wegscheid C., Claass B., Carambia A., Herkel J., Mittrucker H.W., Panzer U., Tiegs G. (2011). CXCR3 deficiency exacerbates liver disease and abrogates tolerance in a mouse model of immune-mediated hepatitis. J. Immunol..

[125] Colvin R.A., Campanella G.S., Manice L.A., Luster A.D. (2006). CXCR3 requires tyrosine sulfation for ligand binding and a second extracellular loop arginine residue for ligand-induced chemotaxis. Mol. Cell. Biol..

[126] Hintermann E., Ehser J., Bayer M., Pfeilschifter J.M., Christen U. (2013). Mechanism of autoimmune hepatic fibrogenesis induced by an adenovirus encoding the human liver autoantigen cytochrome P450 2D6. J. Autoimmun..

[127] Hardtke-Wolenski M., Fischer K., Noyan F., Schlue J., Falk C.S., Stahlhut M., Woller N., Kuehnel F., Taubert R., Manns M.P. (2013). Genetic predisposition and environmental danger signals initiate chronic autoimmune hepatitis driven by CD4+ T cells. Hepatology.

